# The Associations between Meeting 24-Hour Movement Guidelines (24-HMG) and Self-Rated Physical and Mental Health in Older Adults—Cross Sectional Evidence from China

**DOI:** 10.3390/ijerph192013407

**Published:** 2022-10-17

**Authors:** Lin Luo, Yunxia Cao, Yulong Hu, Shaojing Wen, Kaiqi Tang, Lina Ding, Naiqing Song

**Affiliations:** 1 College of Physical Education, Guizhou Normal University, Guiyang 550001, China; 2 Basic Education Research Center, Southwest University, Chongqing 400715, China

**Keywords:** 24-hour movement guidelines, physical activity time, sedentary time, older people, physical and mental health

## Abstract

Background: This study determined the prevalence of older adults (aged 60–80 years) meeting the Canadian 24-HMG alone and in combination, and their association with the self-rated physical health and mental health of older adults. Methods: Participants were drawn from 4134 older adults aged 60–80 years (age, 67.37 ± 0.08 years; 46.87% male) from the China Health and Nutrition Survey (CHNS) 2015 database. Mental health and physical health scores were obtained based on self-rated mental health questionnaires and physical health of older adults. The criteria for meeting the 24-HMG were: physical activity time ≥ 150 min/week, sedentary time ≤ 480 min/day, screen time ≤ 180 min/day, sleep time 7–9 h (60–64 years) and sleep time 7–8 h (65 years and above). Logistic regression models were used to examine the association between meeting the 24-HMG guidelines number and category and older adults’ self-rated mental and physical health. Results: The proportion of older people meeting three of the 24-HMG guidelines was 1.16%, the proportion meeting two guidelines was 38.19% and the proportion meeting one guideline was 49.14%. The number of people meeting 24-HMG was closely related to the self-rated physical health and mental health of older people. The category meeting 24-HMG was closely related to the self-rated physical health and mental health of older people. Conclusions: Meeting more guidelines in the 24-HMG was strongly associated with older people’s self-rated mental health and physical health. However, only a small proportion of older people met all the recommendations, highlighting the need to promote and support adherence to these movement behaviours.

## 1. Introduction

The relationship between adequate physical activity, restricted sedentary time and adequate sleep duration and physical and mental health in older adults has been extensively studied in previous research [1,2,3,4]. Over the course of a 24 h day, changes in the amount of time spent in one movement behaviour will alter the amount of time spent in another. As a result, emerging research has considered how the behaviours interact together to influence health outcomes [5,6]. The Canadian Society for Exercise Physiology (CSEP) has proposed the integration of physical activity, sedentary behaviour and sleep as key components of a 24-hour movement guideline for adults over the age of 18 years and older. In October 2020, the CSEP published the world’s first 24-hour movement guidelines for adults aged 18–64 years and the 24-hour movement guidelines for adults aged 65 years and older [5]. Using the idea of time use of the day [6], researchers proposed a research framework that integrates physical activity time, sedentary time and sleep time, as this combination provides more reliable information on health behaviour change [7]. In 24-HMG, there are three core recommendations for adults: be more physically active, reduce sedentary time and have a good night’s sleep [5].

There is already some research evidence that older adults who consistently performed reasonable physical activity, limited sedentary time and had adequate sleep could achieve additional physical and mental health benefits, such as reduced rates of obesity, type 2 diabetes and metabolic syndrome [8], improved cognitive function [9], and improved health-related quality of life [10].

Previous studies have shown that the three recommendations of adequate physical activity [11,12], restricted sedentary time (or screen time) [13,14] and good sleep duration (to meet age-specific needs) [15,16] in 24-HMG are each associated with physical and mental health in older adults. For example, the results of a meta-analysis by Jakicic et al. supported the fact that physical activity of less than 10 min duration was associated with a variety of health outcomes (body composition, blood pressure, lipids, blood glucose, metabolic syndrome, frailty, all-cause mortality, etc.) [17]. Cunningham et al. found through 24 systematic reviews and meta-analyses that physical activity (PA ≥ 150 min/week) in older people aged 60+ was associated with a reduced risk of all-cause mortality, cardiovascular mortality, breast and prostate cancer, fractures, recurrent falls, ADL disability and functional limitations, cognitive decline, dementia, Alzheimer’s disease and depression [18]. Callow et al. reported that among 1046 older adults aged 50+ years, higher levels of physical activity levels may help to alleviate depressive symptoms in older adults during COVID-19 [19]. Yang and D’Arcy reported that physical activity could reduce the negative impact of chronic illness on mental health and help to improve mental health in older adults (65+ years) [20]. In recent years, a growing number of studies showed that sedentary behaviour was common in older people [21] and increased the risk of morbidity and mortality [22]. Pandey et al. reported a non-linear relationship between questionnaire-assessed sedentary time and cardiovascular morbidity, with an increased risk of CVD observed only in sedentary time greater than 10 h/day. In contrast, in studies using objective assessments of sedentary time, each additional hour of sedentary time increased the risk of CVD by 12% [23]. Several meta-analyses of prospective studies had found that longer sedentary time was associated with an increased risk of death, even after adjustment for physical activity [24]. Richardson et al. reported increased sedentary time in older adults during COVID-19 social isolation and an association between sedentary time and negative health outcomes in older adults [25]. Some studies examined screen time as a separate component of sedentary behaviour. For example, Webb et al. reported that increased screen time in people aged 65+ was associated with their prevalence of anxiety and depression during COVID-19 [26]. Yu et al. reported that ORs for depressive symptoms increased with increased screen time in older adults aged 65+ compared to controls with daily screen time (<1 h/day) [27]. Neophytou et al. reported that increased total screen time was associated with lower self-esteem, mental health problems and premature cognitive decline [28]. A report by Chaput et al. on sleep duration showed that approximately 7–8 h of sleep per day was most favourably associated with the key health outcomes examined [2]. A study by Hirshkowitz et al. reported that long sleep duration (i.e., ≥9–10 h per day) in older adults was associated with comorbidity and mortality, and that excessive sleep may be a marker for the need for medical evaluation [29]. Lücke et al. reported that daily sleep quality was associated with negative affect and stress responses in older adults [30]. Sin et al. reported that the relationship between daily stressors and affective responses to positive events in adults varied according to sleep duration. Sleep deprivation could amplify the loss of positive affect on days with stressors and reduce positive affective responses to positive events [31]. In summary, older people who experienced sufficient time for physical activity, less sedentary time (or screen time) and adequate sleep were likely to have better mental and physical health. Thus, older adults who experienced adequate physical activity time, less sedentary time (or screen time) and adequate sleep might have better mental and physical health.

However, these behavioural studies isolated these three recommendations mainly, and only a few have considered their combined effects on the physical and mental health of adults. For example, Guallar-Castillón et al. reported that any pattern of physical activity included in the Spanish sample was associated with better physical or mental health [32]. Mellow et al.found that more time spent in physical activity, less passive sedentary behaviour and quality sleep were beneficial for cognitive function in older adults [10]. Hofman et al. found that the use of moderate-to-vigorous physical activity instead of sedentary behaviour or sleep was associated with fewer depressive symptoms [33].

With the release of the 24-HMG, some researchers started to examine the meeting of the 24-HMG in adults and its relationship to individual physical and mental health outcomes from the perspective of integrating physical activity time, sedentary time (and/or screen time) and sleep time, rather than examining multiple determinants in isolation. For example, Rollo et al. reported that meeting the 24-hour movement guideline was associated with cardiometabolic, obesity and mortality risk in adults [34]. Liang et al. showed that in a sample of 1846 Chinese university students, meeting sleep guidelines (alone or in combination with other guidelines) was associated with significantly lower levels of depression and anxiety; and that meeting sedentary time and physical activity guidelines was associated with lower levels of depression. Thus, meeting additional guidelines, particularly adherence to healthy sleep habits, may play an important role in promoting mental health in young people [35]. Clarke et al. reported HRs for all-cause mortality for adults meeting none, any one, any two and all three recommendations as 1.00, 0.86, 0.49 and 0.72 [36]. Weatherson et al. found that college students who met the 24-hour exercise guideline had higher mental health scores [37]. Rigotti et al. reported prevalence rates of 48.3%, 22.0%, 19.4% and 1.6% for adults who met the physical activity, sedentary time (≤8 h/day), sleep duration and combined recommendations, respectively [38]. A study by Kastelic et al. found that adults who met sleep guidelines, any combination of two guidelines, or all three guidelines experienced stress less frequently. Meeting the MVPA guidelines alone or in combination with any other motor behaviour guideline was associated with better self-rated health. The likelihood of reduced stress and self-rated health increased as the number of guidelines increased [39]. These studies provided some support for the ability of 24-hour movement to predict physical and mental health in adults.

Several studies also explored the satisfaction [7] and transmission characteristics [40] of 24-HMG in older adults. It has been suggested that integrating overall surveys of physical activity, sedentary time and sleep time may improve their validity in predicting individual health outcomes [5]. Moreover, from a practical application perspective, it could be possible to compare the differences in the impact of different levels of meeting recommendations on the physical and mental health of older people [41]. Although there were numerous studies on the benefits of meeting physical activity time, sedentary time and sleep time on the physical and mental health of older people, and many meta-analyses of evidence were used to develop the adult 24-HMG, the evidence on meeting the 24-HMG to predict self-rated physical and mental health in older people is still very limited. Additionally, older people in different countries may have different preferences for 24-hour movement behaviour, which is related to the socio-economic development and social and cultural background of the region in which they live. There is therefore a strong need to examine the evidence that 24-HMG predicts physical and mental health in older people from different cultural backgrounds to further substantiate the association between 24-HMG and physical and mental health in older people.

In China, most working older people will retire at the age of 60 (it can be 55 for women). Some older people choose to continue working after retirement, and some also help their children with childcare. With the introduction of China’s national fitness programme, many older people have also started to take part in physical exercise after retirement, such as participating in square dancing and tai chi. With the development of information technology and the popularity of the Internet, the screen time of older Chinese people has also shifted from traditional television time to a diverse range of screen time. The aim of this study was to explore the association between satisfying 24-HMG and self-rated physical and mental health in older adults using a sample of Chinese older adults. To our knowledge, this is the first study on the association between 24-HMG and physical and mental health in older adults.

## 2. Materials and Methods

### 2.1. Study Design and Participants

The China Health and Nutrition Survey (CHNS) was an ongoing open cohort international collaboration between the Carolina Population Center at the University of North Carolina at Chapel Hill and the National Institute of Nutrition and Health (NINH, formerly the National Institute of Nutrition and Food). The aim was to examine the effectiveness of health, nutrition and family planning policies and programmes implemented by national and local governments, and to understand how China’s social and economic transformation is socially affecting the health and nutritional status of its population. The survey calendar adopted a multi-stage, random clustering process to sample approximately 7200 households and more than 30,000 individuals in 15 provinces and municipalities directly under the Central Government, which vary widely in terms of geography, economic development, public resources and health indicators. The CHNS questionnaire was started in 1989 and has been followed up every four years. The physical activity component of the survey was introduced in 2004. The age range of the elderly respondents in this study was 60–80 years.

Data for this study was obtained from published survey data from CHNS (2015). The study covered data on demographic characteristics, physical activity time, sedentary time, sleep time and self-rated mental health and stress scores of older adults. The sample size of older adults aged 60–80 years in the 2015 CHNS was 4229. In the 2015 survey, 12 provinces or municipalities in China were surveyed, namely Beijing, Liaoning, Heilongjiang, Shanghai, Jiangsu, Shandong, Henan, Hubei, Hunan, Guangxi, Guizhou and Chongqing. Excluding data on missing mental health scores, physical health scores, physical activity time, sedentary time, and sleep time, the final study sample size for this study was 4134 (46.87% male, mean age = 67.37, SD = 0.08).

For information on the ethical review of this study, please see https://www.cpc.unc.edu/projects/china (accessed on 1 January 2022). Participants signed a consent form. The academic committee of Guizhou Normal University waived ethical review of this study due to the use of secondary publicly available data.

### 2.2. Measures

This study uses the Canadian 24-HMG (for people aged 18–64 and 65 years and older) as a reference standard. There are three recommended goals in 24-HMG. Goal 1 is to be more physically active. This includes a cumulative minimum of 150 min of moderate-- to-vigorous aerobic activity per week, muscle-strengthening activities at least twice a week and several hours of light physical activity, including standing. People over 65 should also include physical activity that challenges balance. Goal 2 is to reduce sedentary time. This includes limiting sedentary time to 8 h or less per day, with no more than 3 h of screen time. Goal 3 is to get a good night’s sleep. Adults aged 18–64 years can sleep up to 7–9 h per day. Adults aged 65 years and over can sleep up to 7–8 h a day. This study focused on meeting the three guidelines in the 24-HMG, encompassing physical activity time, sedentary time and sleep time.

#### 2.2.1. Physical Activity Time

CHNS asked residents about the amount of time they spent exercising in various sports. This study used the total of survey respondents’ daily participation (martial arts, gymnastics, dance, acrobatics, track and field, swimming, football, basketball, tennis, badminton, volleyball, and other physical activities) time from Monday to Sunday. Due to the secondary data used in this study and the limitations of the original questionnaire, strength activity and light physical activity were not specifically investigated in the physical activity survey. Referring to the Canadian 24-HMG, PA ≥ 150 min/week was defined as meeting PA.

#### 2.2.2. Sedentary Time

CHNS asked questions about the various sedentary and screen behaviours experienced by residents. The CHNS collected the amount of time subjects spent enjoying screen activities (watching TV, watching videos, using the online collection only, playing video games, browsing the internet, browsing online, or playing on the computer) from Monday to Sunday. The CHNS also collected the amount of time subjects spent reading books and carrying out other sedentary behaviours from Monday to Sunday. Referring to the Canadian 24-HMG, sedentary time ≤ 480 min/day, along with screen time ≤180 min/day, was defined as meeting ST.

#### 2.2.3. Sleep Time

CHNS used the question “How much sleep time do you get each night?” to obtain self-reported daily sleep duration for older adults. Referring to the Canadian 24-HMG, this study defined meeting sleep as 7–9 h of sleep per night for those aged 60–64 years and 7–8 h of sleep per night for those aged 65 years and older.

#### 2.2.4. Mental Health and Physical Health Scores

Mental health scores: The CHNS assessed mental health using three structured questions on vitality, well-being and optimism: “You have as much energy as you did in 2014”, “You are as happy as you were when you were younger” and “Things are better than you think as you get older”. The three items measure the mental health of older people. Each item was rated on a 5-point Likert scale ranging from 0 = “never” to 4 = “very often”. The Cronbach’s alpha coefficient for this questionnaire was 0.801. The raw scores for the three questions were summed to obtain a total mental health score. There is no accepted cut-off point for mental health scales in the CHNS, and given that most mental health-related scales redefine level and degree in terms of cut-off points, this study was supplemented by using the mean self-rated mental health score (6) as the cut-off point. If the respondent’s self-rated mental health score was less than 6, the respondent was defined as having a low level of mental health and was assigned a value of 0. If the respondent’s self-rated mental health score was greater than or equal to 6, the respondent was defined as having a high level of mental health and was assigned a value of 1.

Physical health scores:The CHNS used the question “How do you think your health is now compared to other people of your age? ”to ask participants about their physical health. The item is rated on a 5-point Likert-type scale ranging from 1 = “very poor” to 5 = “very good”.

#### 2.2.5. Covariates

Study participants were asked to self-report demographic data, including age, gender, residence, education, spousal status, annual income, health insurance, job, BMI, chronic disease, smoking, and drinking. When annual personal income was included in the regression analysis, its logarithmic form was used. Chronic disease was calculated based on the prevalence of hypertension, diabetes, myocardial infarction, stroke, cancer, and asthma from the CHNS survey. BMI was calculated by dividing weight (kg) by the square of height (m) [32]. In this study, WS/T 428-2013 screening criteria for weight determination in adults were used. This is a set of Chinese national standards recommended by the National Health Council of China for adults aged 18 years and above (WS/T 428-2013). BMI is classified as not overweight and overweight or more.

### 2.3. Statistical Analysis

All data analysis was carried out using Stata 17.0 software. Descriptive statistics were used to characterise the sample. Frequency percentages were used for count variables and mean ± SD was used to describe the measured variables. Binary logistic regression analyses and ordered logistic regression analyses were used to test the relationship between the meeting of 24-HMG recommendations of physical activity time (PA ≥ 150 min/week), sedentary time (sedentary time ≤ 480 min/day, along with screen time ≤180 min/day) and sleep time (7–9 h at age 60–64, 7–8 h at age 65 and above) with mental health scores and stress scores. Older adults’ meeting of the guidelines was categorized into eight categories based on the type of guidelines met, including meeting none, only meeting PA, only meeting ST, only meeting sleep, meeting PA + ST, meeting PA + sleep, meeting ST + sleep, and meeting PA + ST + sleep. The number of recommendations for meeting 24-HMG by older people was divided into meeting 0, meeting 1, meeting 2 and meeting 3. All logistic regression models were adjusted for covariates and specifically analysed whether this association was influenced by gender. Total models controlled for age, gender, residence, education, spousal status, annual income, health insurance, job, BMI, chronic disease, smoking, and drinking. Males and females models controlled for age, residence, education, spouse status, annual income, health insurance, job, BMI, chronic disease, smoking, and drinking. Statistical significance was defined as *p* < 0.05.

## 3. Results

### 3.1. Sample Characteristics

The final study sample size for this study was 4134 Chinese older adults (aged 60–80 years). The mean age was 67.37 ± 0.08 years and more participants were female (53.13%) (Table 1). A total of 53.65% of participants self-rated their mental health as good 53.65%, while 32.47% and 9.46% self-rated their physical health as good and very good, respectively. The percentages of frequencies meeting physical exercise time, sedentary time and sleep were 47.20%, 12.93% and 61.99%, respectively. Only 1.61% of participants met all three guidelines in the 24-HMG, 38.19% met any two guidelines, 49.04% met one guideline and 11.16% did not meet any of the guidelines. A description of participant characteristics by gender is shown in Table 1.

### 3.2. Number of 24-HMG Recommendations Met in Relation to Self-Rated Mental Health and Physical Health

The results of the ordered logistic regression analysis of the relationship between the number of 24-HMG recommendations met and self-rated physical health are presented in Table 3. The results of the study showed that the number of 24-HMG recommendations met was closely related to the self-rated physical health of older people. Overall, the ORs were 1.31 (*p* < 0.05), 1.85 and 2.19 (*p* < 0.01) for the meeting 1, meeting 2 and meeting 3 groups, respectively, compared to the meeting 0 group. Compared to the meeting 1 group, the OR in the meeting 2 group was 1.40 *(p* < 0.01). Among males, the OR were 1.43 (*p* < 0.05) and 1.92 (*p* < 0.01) in the meeting 1, and meeting 2 groups compared to the male older adults in the meeting 0 group. Compared to the meeting 1 group, the OR was 1.34 (*p* < 0.01) in the meeting 2 group. Among females, the ORs were 1.15 and 2.99 (*p* < 0.01) for the meeting 2 and meeting 3 groups, respectively, compared to the meeting 0 group of older females. Compared to older women in the meeting 1 group, the OR of the meeting 2 and meeting 3 groups were 1.52 (*p* < 0.01), 2.59 (*p* < 0.05), respectively.

Table 2 presents the results of the relationship between the number of 24-HMG recommendations met and self-rated mental health obtained by binary logistic regression. The results of the study showed that the meeting number of 24-HMG recommendations met was closely related to the mental health of older people. Overall, the odds ratios (OR) in the meeting 2 and meeting 3 group were 1.44 (*p* < 0.01) and 1.81 (*p* < 0.05) when compared to the meeting 0 group. The OR in the meeting 2 group was 1.31 (*p* < 0.01) compared to the meeting 1 group. Among males, the OR in meeting 2 was 1.39 (*p* < 0.01) compared to meeting 1. Among females, compared to Meeting 0, the OR in the meeting 2 and meeting 3 groups were 1.63 and 3.30, respectively (*p* < 0.01). Compared to meeting 1, the OR in the meeting 3 group was 2.48 (*p* < 0.05).

### 3.3. Meeting 24-HMG Categories in Relation to Self-Rated Mental Health and Physical Health

Table 4 presents the results of the relationship between the number of 24-HMG categories met and self-rated mental health obtained by binary logistic regression. The results of the study showed that the number of 24-HMG categories met was closely related to the mental health of older people. Overall, the ORs in the only meeting PA, only meeting ST, and meeting PA + sleep groups were 1.44, 0.51 (*p* < 0.05) and 1.52 (*p* < 0.01), respectively, compared to the meeting none group. Among males, the ORs were 0.45 and 1.44 (*p* < 0.05) in the only meeting ST and meeting PA + sleep groups compared to the meeting none group. Among females, the OR of the only meeting PA, meeting PA + sleep and meeting PA + ST + sleep groups were 1.50, 1.65 and 3.31 respectively (*p* < 0.05) compared to the meeting none group.

Table 5 presented the results of the ordered logistic regression scores for the relationship between the meeting 24-HMG category and self-rated physical health. The results of the study showed that the meeting 24-HMG category was closely related to the self-rated physical health of older people. Overall, compared to the meeting none group, the ORs in the only meeting PA, only meeting sleep, meeting PA + sleep, and meeting PA + ST + sleep groups were 1.37, 1.32 (*p* < 0.05), 1.94 and 2.21 (*p* < 0.05). Among males, the ORs were 1.40, 1.44 (*p* < 0.05) and 2.04 (*p* < 0.01) for the only meeting PA, only meeting sleep, and meeting PA + sleep groups, respectively, compared to the meeting none group. Among women, the ORs were 1.83 and 3.02 (*p* < 0.01) in the meeting PA + sleep, and meeting PA + ST + sleep groups compared to the meeting none group.

## 4. Discussion

This study was based on data from a sample of older adults (aged 60–80 years) from the CHNS (2015) survey to determine the association between the number or category of 24-HMG recommendations met and older adults’ self-rated mental health and physical health. In this study, only 1.61% of participants aged 60–80 years in China were found to meet all three guidelines of the 24-HMG. Although there was no significant difference in the proportion of elderly males and females who met the number of 24-HMG guidelines, there was a significant difference in the proportion of elderly males and females who met the 24-HMG guidelines categories. A total of 53.65% participants rated their self-rated mental health as good 53.65%, while 32.47% and 9.46% rated their self-rated physical health as good and very good, respectively. Meeting more of the guidelines in the 24-HMG was associated with better self-rated mental health and physical health for older adults when analysing the overall sample and the male versus female samples. Significant results for the association between meeting the categories recommended in the 24-HMG, and older people’s self-rated physical health and mental health differed by gender among older people.

In this study, only 1.61% of older people met the three 24-HMG guidelines, indicating a low level of adherence to the guidelines among older people in China. The present study was consistent with the findings of Rigotti et al. for Latin American adults aged 18–54 years. His study found that 1.6% of Latin American adults met the three 24-HMG guidelines [39]. However, it was lower than the findings of Rollo et al. whose study found that the proportion of 8297 Canadian adults aged 18–79 years who met the three 24-HMG guidelines was 7.1% [35]. Analysis of the reasons for the inconsistent results of these studies may be related to the age range of the sample population and the different test instruments selected. However, it is worth noting that the low adherence to 24-HMG among older Chinese people may threaten clinical and public health outcomes. Previous studies carried out have found that the three guidelines of adequate physical activity (PA ≥ 150 min/week) [11,12], restricted sedentary time (sedentary time ≤ 480 min/day, and/or screen time ≤180 min/day) [13,14] and good sleep duration (to meet age-specific needs) [15,16] were each associated with physical and mental health in older adults. Therefore, this issue should be addressed through an effective public health approach. However, there is currently limited evidence on how 24-HMG can be used to optimise health behaviours in older people. Therefore, more studies on the association between 24-HMG compliance and physical and mental health in older people could be conducted in the future to encourage the development of intervention strategies to promote 24-HMG compliance in older people.

Previous studies in this field evaluated systematically the independent effects of physical activity, sedentary (screen) time and sleep on physical and mental health outcomes in older people [11,12,13,14,15,16]. Few studies had evaluated the effects of all three jointly on physical and mental health in older adults. The present study showed that meeting more of the guidelines in the 24-HMG was associated with better self-rated mental health and physical health in older adults. The results of this study highlight the importance of helping older adults meet more of the 24-HMG recommendations, which could be considered as a way to enhance the physical and mental health of older adults in this age group. Previous studies such as that of Kastelic et al. found that adults who met the sleep guidelines, any combination of the two guidelines, or all three guidelines experienced stress less frequently. Meeting the MVPA guidelines alone or in combination with any other movement guideline was associated with better self-rated health. The likelihood of reduced stress and self-rated health increased as the number of guidelines increased [39]. The results of this study suggested that meeting the 24-HMG guidelines was beneficial for maintaining better self-rated mental and physical health in Chinese 60–80 year-olds. These findings may further help refine and update the cross-cultural use of 24-HMG. This study found that meeting one 24-HMG guideline had a significant impact on the OR of self-rated physical health in older people; meeting two 24-HMG guidelines had a significant impact on the OR of self-rated mental health in older people. Although there was no significant difference in the relationship between meeting three guidelines compared to meeting two guidelines in this study, there was no significant difference in the association between meeting three guidelines and older people’s self-rated mental health. However, the findings still suggested that meeting more 24-HMG guidelines was more important for older adults’ self-rated mental health.

An analysis of the categories of meeting 24-HMG guidelines found that meeting the same number of 24-HMG recommendations, but with different content, had different effects on older adults’ self-rated physical and mental health. For example, among those meeting one 24-HMG guideline, only meeting the physical activity time guideline was associated with self-rated physical and mental health in older adults. In contrast, meeting the sedentary time guideline was only associated with older adults’ self-rated mental health, while meeting the sleep time guideline was only associated with older adults’ self-rated physical health. Of the two 24-HMG guidelines, meeting both the physical activity time guideline and the sleep time guideline were only associated with self-rated physical and mental health. This further analysed which specific behavioural combinations were associated with self-rated physical and mental health in older people, which may be useful for implementing more effective interventions. This suggested that differences in these specific behavioural combinations should be taken into account when implementing health behavioural interventions for older people, and targeted promotion and guidance should be provided. If the 24-HMG cannot be met in its entirety, guidance on which behavioural recommendations to meet could be prioritised. Future research should consider the implementation of behavioural change interventions and counselling strategies to promote sustainable health and active lifestyles. Such research could also provide actionable information for the development of evidence-based 24-HMG recommendations, including strategies to achieve them. However, there is currently limited data on the 24-hour movement behaviour of older people in China and recommendations for strategies to enhance active and healthy lifestyles. Such information would be an important contributor to strategic decision-making on health policies.

This study presented empirical evidence from a Chinese sample for the replication of the 24-HMG. Our study would add new research evidence to the field of research promoting physical and mental health promotion among older adults. Using data from the CHNS national sample of older adults, this study measured the association between meeting the physical activity, sedentary time and sleep time recommendations in the 24-HMG and their self-rated mental health and physical health in Chinese older adults aged 60–80 years, using the Canadian 24-HMG as a reference standard. Our findings encouraged the use of an optimal combination of health behaviours to promote mental and physical health in older people. However, there were also limitations to this study. Firstly, the timing of data collection (2015) relative to the release of the 24-HMG (2020) was a limitation. The movement behaviour of the older Chinese population might have changed during this period. The results of this study need to be further validated using data from other large samples of Chinese older adults. Secondly, physical activity time, sedentary time, and sleep time were all derived from subjective self-reports and were not objectively measured. In addition, the CHNS did not assess physical activity time exactly as recommended by the 24-HMG. Thirdly, the instruments used in this study to measure the mental health and physical health of older people were not derived from commonly used standardised mental health and physical health measurement instruments. Although the validity of the CHNS measure of older people’s mental health has been validated in a number of studies [42], we were unable to obtain additional evidence of data from its development. Fourthly, the control covariates selected for this study were small, with only age, gender, residence, education, spousal status, annual income, health insurance, job, BMI, chronic disease, smoking, and drinking being controlled for. Finally, due to the cross-sectional design used in this study, no conclusions could be drawn regarding causality. Therefore, further longitudinal studies could be designed for further exploration of the relationship in future studies. On the other hand, the present study had several strengths. Firstly, to the authors’ knowledge, this was the first study to describe the level of meeting the 24-HMG recommendations and the association of self-rated physical and mental health in older Chinese people. Secondly, the use of standardised survey methods for participation by older people from 12 Chinese provinces and cities was an advantage.

## 5. Conclusions

Meeting one guideline (physical activity time), meeting two guidelines (meeting PA + sleep) and meeting three guidelines (meeting PA + ST + sleep) were all associated with significantly better self-rated mental health and physical health in older people compared to not meeting any of the 24-HMG recommendations. Overall, meeting more of the 24-HMG recommendations was associated with better self-rated mental health and physical health outcomes in older adults. However, only a small proportion of older adults met all recommendations, highlighting the need to promote and support adherence to these health behaviours.

## Figures and Tables

**Table 1:** Characteristics of older people in China.

	Mean ± SD/%			t/X^2^	*p*-Value
	Total	Male	Female		
Age (y)	67.37 ± 0.08	67.35 ± 0.12	67.38 ± 0.11	0.190	0.847
Residence				1.240	0.265
City	48.04	48.97	47.23		
Rural	51.96	51.03	52.77		
Education				31.665	**<0.001**
Primary school and below	33.62	30.01	38.04		
Middle school	34.47	36.01	32.57		
High/Higher Vocational/Secondary School	22.30	22.34	22.25		
College and above	9.61	11.63	7.14		
Spousal status				1.033	0.309
None	1.18	1.36	1.02		
Yes	98.82	98.64	98.98		
Annual income (RMB)	15,262.24 ± 687.77	14,875.27 ± 1004.738	15,603.58 ± 943.63	0.530	0.597
Health insurance				0.990	0.320
None	4.42	4.09	4.72		
Yes	95.58	95.91	95.28		
Job				106.449	**<0.001**
None	82.34	75.8	88.03		
Yes	17.66	24.12	11.97		
BMI				8.751	**0.003**
Not overweight	43.46	45.86	41.34		
Overweight and above	56.54	54.14	58.66		
Chronic disease				0.229	0.632
None	67.39	67.76	67.07		
Yes	32.61	32.24	32.93		
Smoking				1.303	**<0.001**
None	73.69	47.70	96.50		
Yes	26.31	52.30	3.50		
Drinking				956.230	**<0.001**
None	76.30	54.37			
Yes	23.70	95.55			
Meeting PA				0.805	0.370
No	52.80	52.07	53.45		
Yes	47.20	47.93	46.55		
Meeting ST				18.124	**<0.001**
No	87.07	89.40	85.00		
Yes	12.93	10.60	15.00		
Meeting Sleep				2.273	0.132
No	29.89	28.76	30.89		
Yes	69.11	71.24	69.11		
Meeting 24-HMG category				21.557	**0.003**
Meeting none	11.16	11.25	11.08		
Only meeting PA	12.56	12.36	12.73		
Only meeting ST	5.46	4.69	6.14		
Only meeting sleep	31.02	31.84	30.31		
Meeting PA + ST	0.71	0.45	0.93		
Meeting PA + sleep	32.32	33.96	30.89		
Meeting ST + sleep	5.15	4.29	5.92		
Meeting PA + ST + sleep	1.61	1.16	2.00		
Meeting number of 24-HMG				4.944	0.176
Meeting 0	11.16	11.25	11.08		
Meeting 1	49.04	48.89	49.18		
Meeting 2	38.19	38.70	37.74		
Meeting 3	1.61	1.16	2.00		
Mental health				11.724	**0.001**
None	46.35	49.14	43.88		
Yes	53.65	50.86	56.12		
Physical health				18.352	**0.001**
Very poor	1.96	1.51	2.36		
Poor	9.96	8.53	11.21		
Moderate	46.16	45.71	46.55		
Good	32.47	33.65	31.42		
Very good	9.46	10.60	8.46		

Sleep: sleep guidelines; ST: sedentary time guidelines; PA: physical activity guidelines. *p*-values in bold indicate significant levels < 0.05.

**Table 2:** The relationship between the number of 24-HMG recommendations met and mental health in the sample.

	Total		Male		Female	
	OR (95% CI)	*p*-Value	OR (95% CI)	*p*-Value	OR (95% CI)	*p*-Value
Ref:Meeting 0						
Meeting 1	1.09 (0.84~1.42)	0.487	0.94 (0.67~1.34)	0.770	1.32 (0.87~2.01)	0.185
Meeting 2	1.44 (1.06~1.89)	**0.007**	1.32 (0.92~1.88)	0.122	1.63 (1.07~2.49)	**0.022**
Meeting 3	1.81 (0.93~3.50)	**0.049**	0.87 (0.32~2.31)	0.786	3.30 (1.25~8.69)	**0.016**
Ref:Meeting 1						
Meeting 2	1.31 (1.11~1.55)	**0.001**	1.39 (1.12~1.74)	0.004	1.23 (0.49~1.14)	**0.089**
Meeting 3	1.64 (0.88~3.08)	0.118	0.91 (0.36~2.34)	0.861	2.48 (1.01~6.16)	**0.049**
Ref:Meeting 2						
Meeting 3	1.24(0.66~2.33)	0.486	0.66(0.25~1.68)	0.387	2.01 (0.81~4.99)	0.130

Ref: reference group; *p*-values in bold indicate significant levels < 0.05.

**Table 3:** The relationship between the number of 24-HMG recommendations met and physical health in the sample.

	Total		Male		Female	
	OR (95% CI)	*p*-Value	OR (95% CI)	*p*-Value	OR (95% CI)	*p*-Value
Ref:meeting 0						
Meeting 1	1.31 (1.02~1.68)	**0.029**	1.43 (1.03~1.97)	**0.029**	1.15 (0.78~1.69)	0.458
Meeting 2	1.85 (1.44~2.38)	**<0.001**	1.92 (1.38~2.67)	**<0.001**	1.76 (1.19~2.60)	**0.004**
Meeting 3	2.19 (1.21~3.96)	**0.009**	1.28 (0.51~3.20)	0.588	2.99 (1.34~6.65)	**0.007**
Ref:meeting 1						
Meeting 2	1.40 (1.21~1.63)	**<0.001**	1.34 (1.09~1.65)	**0.005**	1.52 (1.22~1.90)	**<0.001**
Meeting 3	1.66 (0.95~2.91)	0.072	0.89 (0.37~2.15)	0.812	2.59 (1.24~5.38)	**0.011**
Ref:meeting 2						
Meeting 3	1.18 (0.67~2.06)	0.553	0.66 (0.27~1.60)	0.369	1.69 (0.82~3.51)	0.154

Sleep: sleep guidelines; ST: sedentary time guidelines; PA: physical activity guidelines; OR: odd ratio; CI: confidence interval. Ref: reference group. *p*-values in bold indicate significant levels < 0.05.

**Table 4:** The relationship between meeting 24-HMG categories and mental health in the sample.

	Total		Male		Female	
	OR (95% CI)	*p*-Value	OR (95% CI)	*p*-Value	OR (95% CI)	*p*-Value
Ref:meeting none						
Only meeting PA	1.44 (1.05~1.97)	**0.023**	1.41 (0.92~2.16)	0.110	1.50 (0.92~2.42)	**0.027**
Only meeting ST	0.51 (0.27~0.96)	**0.039**	0.45 (0.18~1.16)	**0.012**	0.61 (0.25~1.49)	0.283
Only meeting sleep	1.01 (0.76~1.33)	0.923	0.83 (0.58~1.20)	0.333	1.31 (0.84~2.03)	0.229
Meeting PA + ST	1.96 (0.42~9.13)	0.387	4.54 (0.45~45.40)	0.198	0.72 (0.06~8.50)	0.801
Meeting PA + sleep	1.52 (1.16~2.01)	**0.002**	1.44 (1.01~2.07)	**0.046**	1.65 (1.08~2.53)	**0.021**
Meeting ST + sleep	1.05 (0.66~1.67)	0.811	0.73 (0.39~1.37)	0.342	1.65 (0.83~3.28)	0.153
Meeting PA + ST + sleep	1.83 (0.94~3.55)	0.072	0.88 (0.33~2.34)	0.810	3.31 (1.25~8.71)	**0.015**

Sleep: sleep guidelines; ST: sedentary time guidelines; PA: physical activity guidelines; OR: odd ratio; CI: confidence interval. Ref: reference group. *p*-values in bold indicate significant levels < 0.05.

**Table 5:** The relationship between meeting 24-HMG categories and physical health in the sample.

	Total		Male		Female	
	OR (95% CI)	*p*-Value	OR (95% CI)	*p*-Value	OR (95% CI)	*p*-Value
Ref:meeting none						
Only meeting PA	1.37 (1.02~1.84)	**0.032**	1.40 (0.79~2.17)	**0.024**	1.26 (0.81~1.97)	0.292
Only meeting ST	0.81 (0.46~1.44)	0.485	1.19 (0.51~2.76)	0.672	0.58 (0.72~1.27)	0.177
Only meeting sleep	1.32 (1.02~1.71)	**0.031**	1.44 (1.02~2.01)	**0.034**	1.16 (0.77~1.74)	0.465
Meeting PA + ST	2.59 (0.61~11.01)	0.197	5.1 (0.70~26.79)	0.105	1.42 (0.16~12.23)	0.744
Meeting PA + sleep	1.94 (1.50~2.51)	**<0.001**	2.04 (1.46~2.86)	**0.001**	1.83 (1.23~2.72)	**0.003**
Meeting ST + sleep	1.24 (0.81~1.91)	0.307	1.10 (0.62~1.96)	0.729	1.40 (0.74~2.63)	0.295
Meeting PA + ST + sleep	2.21 (1.22~4.00)	**0.009**	1.29 (0.52~3.23)	0.574	3.02 (1.36~6.72)	**0.007**

Sleep: sleep guidelines; ST: sedentary time guidelines; PA: physical activity guidelines; OR: odd ratio; CI: confidence interval. Ref: reference group. *p*-values in bold indicate significant levels < 0.05.

## Data Availability

Data can be requested.
